# High-Dose Irradiation Inhibits Motility and Induces Autophagy in *Caenorhabditis elegans*

**DOI:** 10.3390/ijms22189810

**Published:** 2021-09-10

**Authors:** Akira Yamasaki, Michiyo Suzuki, Tomoo Funayama, Takahito Moriwaki, Tetsuya Sakashita, Yasuhiko Kobayashi, Qiu-Mei Zhang-Akiyama

**Affiliations:** 1 Laboratory of Stress Response Biology, Graduate School of Science, Kyoto University, Kitashirakawa-Oiwakecho, Sakyo-ku, Kyoto 606-8502, Japan; yamasakiakira8@hotmail.co.jp (A.Y.); moriwaki@med.kawasaki-m.ac.jp (T.M.); 2 Department of Radiation-Applied Biology Research, Takasaki Advanced Radiation Research Institute, National Institutes for Quantum and Radiological Science and Technology (QST-Takasaki), 1233 Watanuki, Takasaki 370-1292, Japan; funayama.tomo@qst.go.jp (T.F.); sakashita.tetsuya@qst.go.jp (T.S.); kobayashi.yasuhiko@qst.go.jp (Y.K.); 3 Department of Molecular and Genetic Medicine, Kawasaki Medical School, Okayama 701-0192, Japan

**Keywords:** region-specific irradiation, microbeam, locomotion, swimming, autophagy, *C. elegans*

## Abstract

Radiation damages many cellular components and disrupts cellular functions, and was previously reported to impair locomotion in the model organism *Caenorhabditis elegans*. However, the response to even higher doses is not clear. First, to investigate the effects of high-dose radiation on the locomotion of *C. elegans*, we investigated the dose range that reduces whole-body locomotion or leads to death. Irradiation was performed in the range of 0–6 kGy. In the crawling analysis, motility decreased after irradiation in a dose-dependent manner. Exposure to 6 kGy of radiation affected crawling on agar immediately and caused the complete loss of motility. Both γ-rays and carbon-ion beams significantly reduced crawling motility at 3 kGy. Next, swimming in buffer was measured as a motility index to assess the response over time after irradiation and motility similarly decreased. However, swimming partially recovered 6 h after irradiation with 3 kGy of γ-rays. To examine the possibility of a recovery mechanism, in situ GFP reporter assay of the autophagy-related gene *lgg-1* was performed. The fluorescence intensity was stronger in the anterior half of the body 7 h after irradiation with 3 kGy of γ-rays. GFP::LGG-1 induction was observed in the pharynx, neurons along the body, and the intestine. Furthermore, worms were exposed to region-specific radiation with carbon-ion microbeams and the trajectory of crawling was measured by image processing. Motility was lower after anterior-half body irradiation than after posterior-half body irradiation. This further supported that the anterior half of the body is important in the locomotory response to radiation.

## 1. Introduction

Living organisms are frequently exposed to radiation. On Earth, we are exposed not only to natural radiation, but also to artificial radiation, such as γ rays and X-rays, from medical devices and nuclear accidents. In addition to X-rays and γ rays, there are several types of particle radiation in space such as neutrons and heavy ions. Radiation damages living organisms by directly affecting cellular components with its energy. In addition, there are indirect damaging effects of reactive oxygen species (ROS) produced by the ionization of water molecules in the cells by radiation. Sensitivity to radiation varies among species. In humans and mice, irradiation of 4–7 Gy is usually fatal, resulting in death of 50% of individuals 1–2 months after exposure (half-lethal dose) [1]. Some animals are also highly radioresistant. The 10% lethal dose for *Adineta vaga* (tetraploid rotifer) is 1200 Gy [2]. The half-lethal dose for *Milnesium tardigradum* (tardigrades) is 5000 Gy [3]. Prokaryotes are also resistant to higher doses of radiation. For example, *Escherichia coli* has a 10% survival rate at 700 Gy and *Deinococcus radiodurans* survives after 12,000 Gy [3].

The effects of radiation vary from tissue to tissue, with mitotic cells being more sensitive [4,5,6]. Non-dividing cells, such as neural and cardiomyocyte cells, are less sensitive to radiation and are affected by high exposure doses [7,8,9]. In such cases, these effects are unclear, but they may lead to motor function disorders and memory impairment.

An adult *Caenorhabditis elegans* (*C. elegans*) hermaphrodite consists of 959 somatic cells, and has neural, muscular, intestinal, and gonads tissues. It has dividing gonadal cells and non-dividing somatic cells [10,11,12]. *C. elegans* is used as a model organism because its body is transparent and its internal structure can be observed. In radiation biology, the effects of radiation on vital functions, such as development, locomotion, learning, and aging, have been examined using *C. elegans* [13]. In adult dividing gonadal cells in an adult *C. elegans*, exposure to 0.1 kGy of carbon ions induces germ cell corpses and the eggs do not hatch [14]. When the eggs were irradiated with 0.1 kGy of carbon ions, the hatchability was approximately 10% [15]. The radiosensitivity of individual *C. elegans* was less than that for 5 Gy at 4 h after hatching, and the 37% average lethal dose for second larvae (L2) was approximately 500 Gy [16]. On the other hand, the adult *C. elegans* remained alive even after whole-body exposure to 1 kGy of γ-rays [17]. High-dose irradiation causes a temporary decrease in locomotion [18]. Body bends (head bends for 20 s) were significantly reduced when the whole body was irradiated with 0.5 kGy of carbon ions [19]. Irradiation with 1 kGy of carbon beams reduced the locomotion [15,18,19]. In this manner, non-dividing and dividing cells are different in their radiosensitivity. In addition, the sensitivity to oxidative stress is different between non-dividing and dividing stages [20]. However, the mechanisms of these effects are not fully understood. Furthermore, knowledge regarding responses of *C. elegans* irradiated with a high dose of over 1.5 kGy is very limited [13].

Autophagy is a mechanism for degrading proteins and intracellular organelles, and producing amino acids as degradation products [21,22,23]. Autophagy is caused by starvation and exercise, and this control affects glucose metabolism and the control of motor function [24]. Autophagy is induced by oxidative stress in *C. elegans* [20,25] and in germ cells by radiation [25,26]. ROS cause non-specific oxidative damage to numerous molecules and organelles in the cell. Whether autophagy is induced in neurons and muscle cells involved in motor function is of interest.

Region-specific irradiation techniques using microbeams have been established [27,28], and an experimental system of localized microbeam irradiation of *C. elegans* without anesthesia to examine motor function was previously reported [29,30]. Local irradiation experiments using microbeams affected the motility of the head, abdomen, and tail [28]. However, microbeam irradiation targeted to the central nervous system (CNS) had limited effects on motor function [27]. Although the tissues that are important for reducing motility have not yet been identified, region-specific microbeam irradiation techniques make it possible to investigate the effects of irradiation on each part of the tissue of *C. elegans*.

In this study, we examined the effects of high-dose radiation on the motility of *C. elegans* by comparing γ-rays and carbon-ion beams, and found that both types of radiation reduce the motility. Moreover, motor function recovered after irradiation. Autophagy was observed using GFP::LGG-1, and was induced in the pharynx, nerve cells (neurons) along the body, and the intestine after irradiation. Furthermore, we conducted carbon-ion microbeam irradiation of somatic cells to investigate which tissues are important for motor function. Our study suggested that there are important tissues in the anterior half of the body.

## 2. Results

### 2.1. Motility Was Reduced by Whole-Body Irradiation with High-Dose γ-rays and Carbon-Ion Beams

Previous studies reported that the locomotion of adult worms was reduced by 1420 Gy of γ-ray irradiation [19] or 1 kGy of carbon-ion beam irradiation [17]. However, little is known about the effects of higher doses on locomotion. To investigate the effects of higher doses, γ-rays and carbon-ion beams were used to detect the influence on motor function immediately and 24 h after irradiation. After irradiation by 0.5 kGy, no eggs hatched in this study (data not shown).

**Figure 1:**
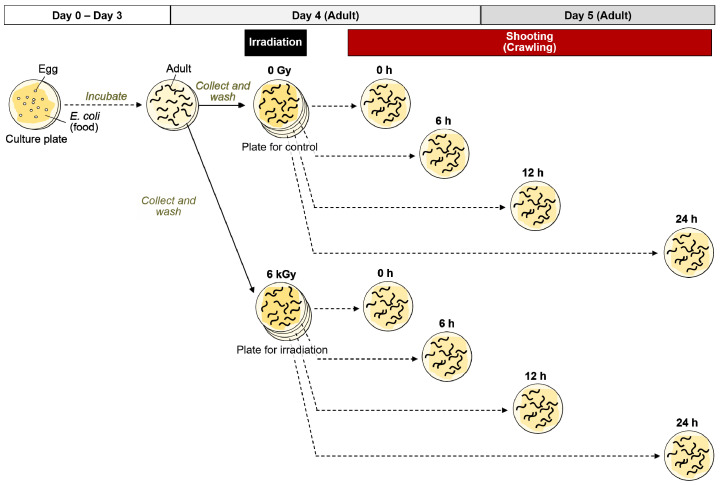
Procedure for time-course crawling assay of *C. elegans* after γ-ray irradiation. Well-fed adult worms were irradiated with a dose of 6 kGy of γ-rays on a NGM plate spread with a bacterial lawn. At 0, 6, 12, and 24 h after irradiation, the crawling of 5 worms, which were randomly selected from worms, were video-recorded, respectively. In the same way, non-irradiated control worms were video-recorded.

**Figure 2:**
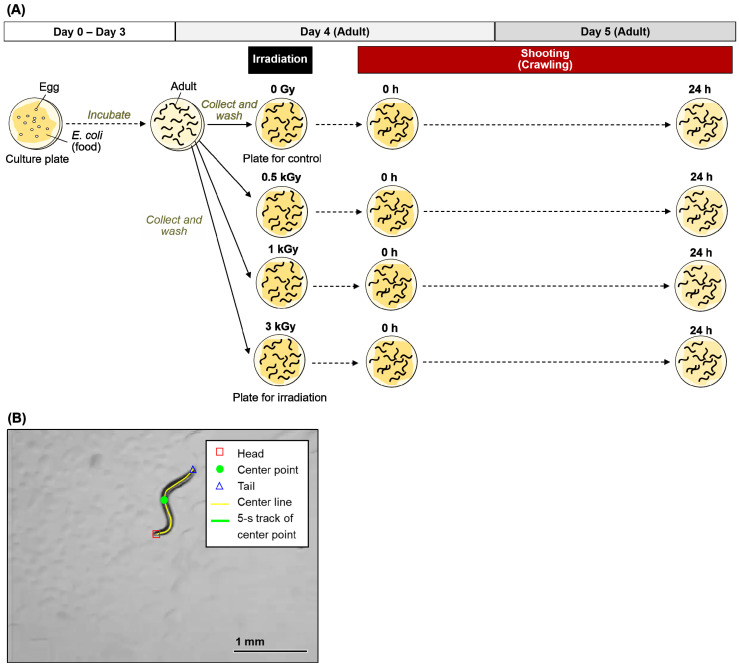
Procedure for crawling assay of *C. elegans* after γ-ray or carbon-ion whole-body irradiation. (**A**) The scheme of this experiment. Well-fed adult worms were irradiated with γ-rays or carbon-ion beams. After irradiation, the crawling of 5 worms, which were randomly selected from worms on NGM plates spread with a bacterial lawn, were video-recorded. The procedure was conducted through independent irradiation trials, i.e., 6 irradiation trials for γ-rays and 4 trials for carbon-ion beams, respectively. Thirty worms were tested in each condition in 6 trials of γ-rays irradiation (5 worms per condition per trial). Twenty worms were tested in each condition in 4 trials of carbon-ion irradiation (5 worms per condition per trial). (**B**) An example of image processing for analysis of crawling in *C. elegans* on agar. This image is a first scene (0 s) of forward motion of a non-irradiated (0 Gy) wild-type worm, and the red square, green circle, blue triangle, and yellow line represent the head, center point, tail, and center line, respectively. The green line shows the 5-s track length of center point. Values derived by image processing are as follows: mean worm length, which is mean of center line length for 5 s, was 1.185 mm; 5-s track length of center line was 2.194 mm; and 5-s track length/mean worm length was 1.85.

**Figure 3:**
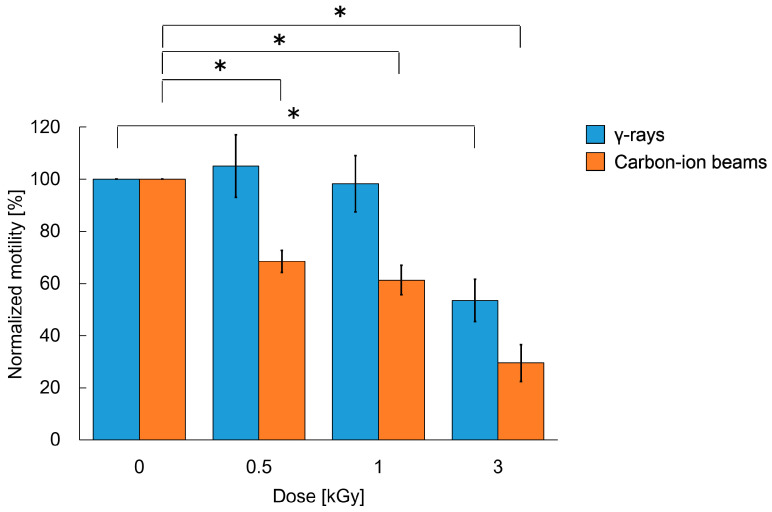
Crawling ability of *C. elegans* immediately after whole-body irradiation with different doses of γ-rays and carbon ion beams. Normalized value of motility of worms immediately (at 0 h) after whole-body irradiation compared with the non-irradiated worms. Track length (trajectory) of the center point of the body in crawling on agar for 5-s was derived from a video, as shown in Figure 2B, and the value was divided by the worm length. In both cases of γ-rays and carbon-ion beam irradiation, the values of 5 worms were averaged at each dose, and the means of worms irradiated with each dose (0, 0.5, 1, and 3 kGy) immediately after irradiation (at 0 h) were divided by each mean value of the corresponding non-irradiated control worms (0 Gy at 0 h). By using this normalized value representing the ratio of motility in crawling on agar, the effects of irradiation on motility can be exactly evaluated. Thirty worms were tested in each condition in 6 trials of γ-rays irradiation (5 worms per condition per trial). Twenty worms were tested in each condition in 4 trials of carbon-ion-beams irradiation (5 worms per condition per trial). The mean values of the normalized motility of 6 or 4 irradiation trials are presented in blue or orange, respectively. Error bar indicates SEM. The data were analyzed by the Student’s *t*-test. * indicates *p-*value < 0.05, significantly different from 0 Gy.

Based on the procedures for the crawling assay shown in Figure 1 and Figure 2A, adult worms were transferred to the nematode growth medium (NGM) plates spread with a bacterial lawn and irradiated with 0 to 6 kGy of γ-rays or carbon ion beams. Crawling on an agar plate spread with bacterial lawn (food) was video-recorded immediately after irradiation (at 0 h) (Figure 2B, Video S1). The crawling was recorded for 10 s. The moved distance at the center point of body for 5 s (5 s track length) and the mean length of the center line of the body were derived using a worm-specific image processing software (Figure 2B) [31]. We confirmed that there was little difference between the first 5 s and the second 5 s of the motility (data not shown). To evaluate the effects of irradiation on motility we calculated ‘the normalized motility’ using the derived values from videos as described in detail below (see Section 4.3). We first investigated the effects of 6 kGy irradiation based on the time-course assay as shown in Figure 1. Motility of crawling on agar was inhibited and completely stopped by irradiation with either of 6 kGy of γ-rays (Video S2) or 6 kGy of carbon ion beams (data not shown). We observed motility at 0, 6, 12, and 24 h, but motility did not recover (data not shown). Next, the dose dependency using 0, 0.5, 1, or 3 kGy was investigated. A dose-dependent decrease in motility of crawling on agar with food was observed when the worms were irradiated with 3 kGy of γ-rays or carbon ion beams (Figure 3). The motility was reduced to 54% by 3 kGy γ-ray irradiation. Similarly, irradiation with 3 kGy of carbon-ion beams reduced the motility to 30%.

### 2.2. The Decreased Motility after Whole-Body Irradiation Partially Recovered

**Figure 4:**
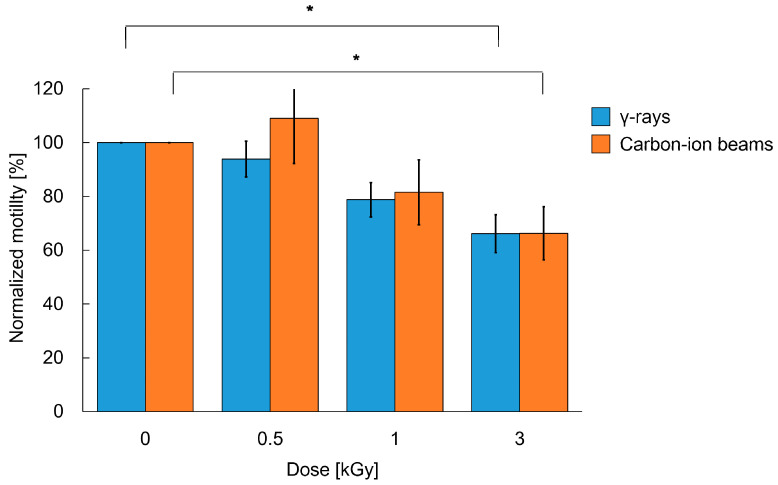
Crawling ability of *C. elegans* 24 h after whole-body irradiation with different doses of γ-rays and carbon ion beams. Normalized value of motility of worms 24 h (one day) after irradiation. Track length (trajectory) of the center point of the body in crawling on agar for 5-s was derived from a video based on the same procedure shown in Figure 3. Thirty worms were tested in each condition in 6 trials of γ-rays irradiation (5 worms per condition per trial). Twenty worms were tested in each condition in 4 trials of carbon-ion-beams irradiation (5 worms per condition per trial). The mean values of the normalized motility of 6 or 4 irradiation trials are presented in blue or orange, respectively. Error bar indicates SEM. The data were analyzed by the Student’s *t*-test. * indicates *p-*value < 0.05, significantly different from 0 Gy.

The whole-body irradiated worms were incubated and then observed again at 24 h (one day) after irradiation. The motility of worms 24 h after 6 kGy of whole-body γ-ray irradiation did not recover (Video S2), whereas the worms irradiated with 0.5, 1, or 3 kGy of carbon-ion beams moved more actively than those immediately after irradiation (0 h) (Figure 4). After 3 kGy of γ-ray irradiation, the motility of worms recovered to 66%. On the other hand, after carbon-ion beam irradiation, the motility of worms recovered from 68% to 109% of the non-irradiated worms at 0.5 kGy, from 61% to 82% of the non-irradiated worms at 1 kGy, and after 3 kGy, motility recovered up to 66% of the non-irradiated worms. Thus, we confirmed that the crawling of adult *C. elegans* on agar can recover at some extent at 24 h after whole-body irradiation with either γ-rays or carbon ion beams (up to a dose of 3 kGy).

**Figure 5:**
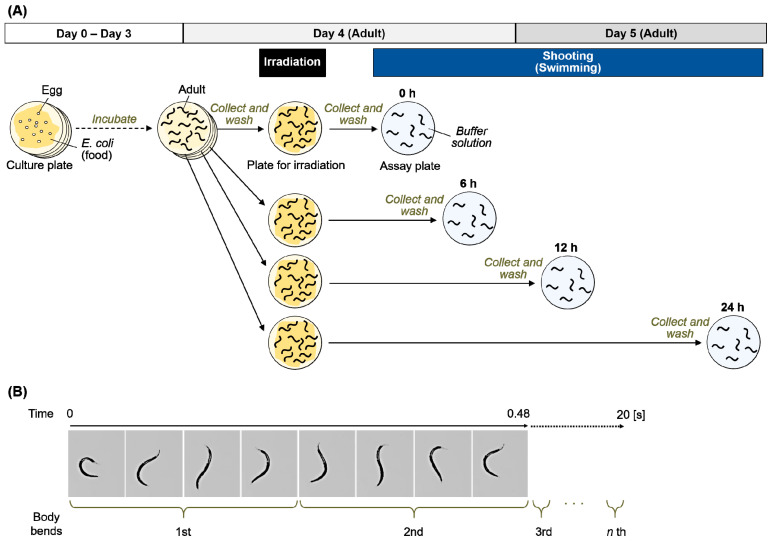
Procedure for swimming assay of *C. elegans* after whole-body γ-ray irradiation. (**A**) Scheme of this experiment. Well-fed adult worms were irradiated with γ-rays. After irradiation, the worms were incubated for 0, 6, 12, and 24 h, transferred to the NGM plate, and S-basal buffer solution was added. Swimming behavior of worms was video-recorded for at least 20 s. (**B**) The number of head swings of a worm in S-basal buffer solution for 20 s was counted manually based on the movie.

We next focused on motility in another condition, in S-basal buffer solution, and evaluated swimming of irradiated worms. Based on the procedures for the swimming (fast thrashing motion) assay shown in Figure 5A, swimming was evaluated from immediately after irradiation to 24 h later. To conduct time course observation, 4 NGM plates with worms cultured on food were irradiated with 3 kGy of γ-rays, and then worms were cultured for 0, 6, 12, or 24 h after whole-body irradiation at 20 °C. After incubation, the worms were transferred to a fresh NGM plate without food, and 3 mL or more of S-basal buffer solution was added into the plate. The swimming irradiated worms were video-recorded for at least 30 s. Moriwaki et al. [32] and Gaffney et al. [33] previously reported that worm motility can be evaluated by counting the frequency of sinusoidal movements for 20 s. As shown in Figure 5B, the number of head swings of worms for 20 s was measured at 0, 6, 12, and 24 h.

**Figure 6:**
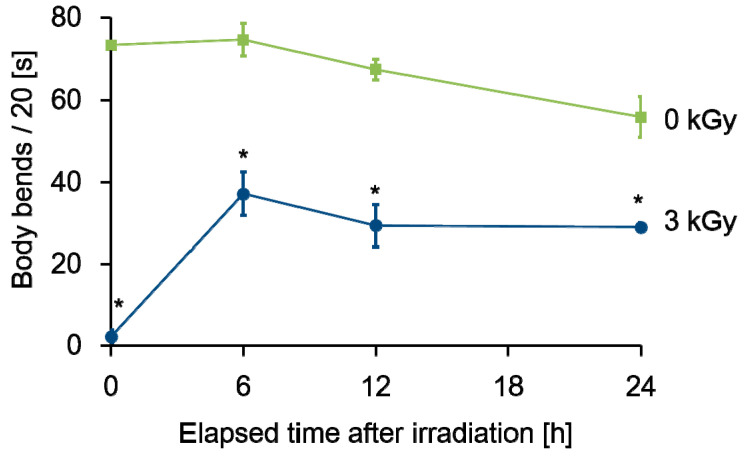
Time course of swimming ability of *C. elegans* after whole-body γ-ray irradiation. Head swing frequency, denoting body bends per 20 s, in S-basal buffer solution of 10 worms was calculated at each time based on the procedure shown in Figure 5B and averaged. The mean of 3 independent experiments is shows as a square for the non-irradiated control worms and as a circle for the γ-ray irradiated worms at 3 kGy, respectively. Error bar indicates SEM. The data were analyzed by the Student’s *t*-test. * indicates *p-*value < 0.05, significantly different from 0 Gy.

As a result, the number of head swings of non-irradiated worms was around 70, and the frequency was slightly decreased from 0 to 24 h after irradiation (green line in Figure 6). In contrast, worms irradiated with 3 kGy of γ-rays lost the head-swing ability immediately after irradiation and the number of head swings was 0 (blue line in Figure 6). Six hours after irradiation, the value recovered to approximately 30, and was maintained until 24 h after irradiation. The partial recovery of irradiation-induced reduction of motor function suggested that there are some radiation response mechanisms that affected motor function.

### 2.3. Autophagy Was Induced during the Recovery of Decreased Motility after Whole-Body Irradiation

Radiation causes not only DNA damage, but also the damage of proteins and intracellular organelles such as mitochondria [34,35,36]. Abnormal protein and organelle accumulation are considered a reason for disturbed cellular function [21,37]. Autophagy is one of the mechanisms for the degradation of abnormal proteins and non-functional mitochondria [38,39]. Mitochondrial function is important for maintaining motor function [40,41]. Autophagy has the ability to remove abnormal mitochondria [42]. We previously reported that treatment of adult *C. elegans* with oxidant agents, such as H_2_O_2,_ induces autophagy [20]. At neutral pH and room temperature, 0.035 mM H_2_O_2_ was produced by 500 Gy of ^60^Co γ-rays and 0.049 mM H_2_O_2_ was produced by 500 Gy of high-LET irradiation (100 keV/μm) [43,44]. This suggests that the ROS generated by radiation can induce autophagy. We next examined whether autophagy was induced during locomotion recovery.

**Figure 7:**
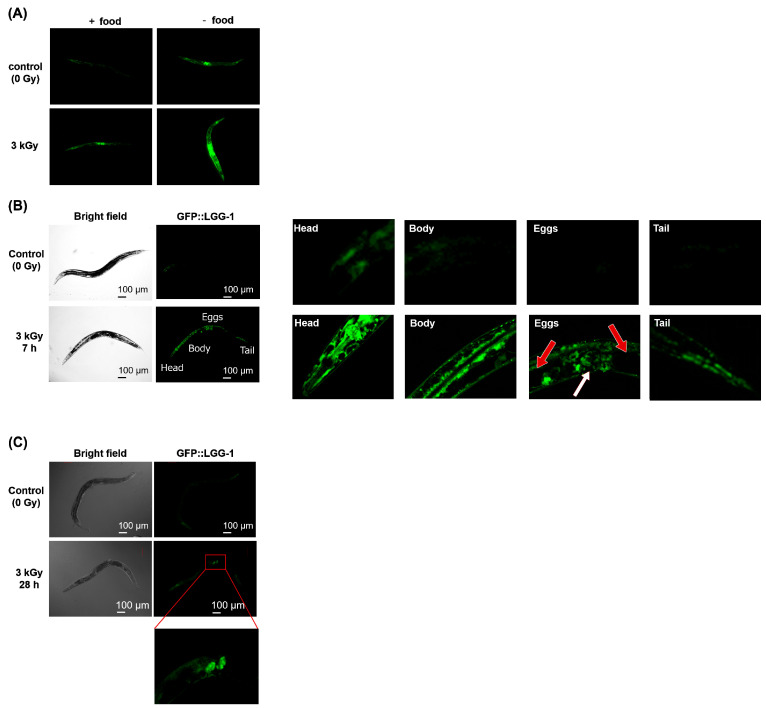
Detection of autophagy after whole-body γ-ray irradiation by GFP::LGG-1 assay. Well-fed adult DA2123 [*lgg-1*p::*gfp*::*lgg-1* + *rol-6*(*su1006*)] *C. elegans* were irradiated with 3 kGy of γ-rays to the whole body. After incubation in the dark at 20 °C on NGM plates with a bacterial lawn, worms were paralyzed using sodium azide and mounted on an agar pad. Then, GFP fluorescence from the worms was observed by microscopy using a 488 nm argon laser. (**A**) Fluorescence in worms 12 h after γ-ray irradiation with or without food. (**B**) Fluorescence in worms 7 h after γ-ray irradiation. An irradiated worm was enlarged to show the head, anterior body, eggs, and tail. The white arrow indicates the vulva and red arrows indicate eggs. (**C**) Fluorescence in worms 28 h after γ-ray irradiation.

To detect autophagy, the LGG-1 (LC3/ATG8 homolog of *C. elegans*) GFP reporter strain DA2123 [*lgg-1*p::GFP::*lgg-1* + *rol-6* (*su1006*)] was used [45,46]. DA2123 adults were irradiated with 3 kGy of γ-rays and cultured in the dark at 20 °C for 28 h with *E. coli.* We observed GFP fluorescence by fluorescence microscopy during incubation and the strongest fluorescence signal was observed at around 6-to-7 h. In our previous report, we treated worms with H_2_O_2_ and examined the expression of GFP::LGG-1 after 6-to-7-hour treatment using approximately 150 worms [20]. In this study, to clarify which tissue responds to irradiation, we investigated the expression pattern in each individual body by observing the GFP::LGG-1 fluorescence. We measured 3 worms in each experiment and repeated 3 times. Consistent with previous studies [47,48], fluorescence was observed under food starvation conditions. Furthermore, fluorescence increased after irradiation with 3 kGy (Figure 7A). The exposure was adjusted such that the expression site was observable. Fluorescence was observed throughout the body of irradiated worms and the fluorescence signal was 2.5-times stronger than that in non-irradiated worms (Figure 7B). The fluorescence intensity was approximately 1.5-times higher in the anterior half of the body than in the posterior half.

The organs that respond to radiation were also examined using fluorescence microscopy. Enlargement of different parts of the body (Figure 7B) corresponded to strong fluorescence in the pharynx, as shown in Figure 7B, and strong fluorescence was also observed in the intestine and the neurons along the body wall. No fluorescence was observed around the eggs (Figure 7B, red arrow), but weak fluorescence was observed around the vulva (white arrow). As shown in Figure 7B, fluorescence was observed in the intestine. Thus, autophagy was induced in the pharynx, neurons along the body, and intestine by irradiation. After 28 h, the fluorescence disappeared in somatic cells, but was observed in some eggs (Figure 7C).

### 2.4. Anterior Half-Body Irradiation by Carbon ion Microbeams Affects Motility

**Figure 8:**
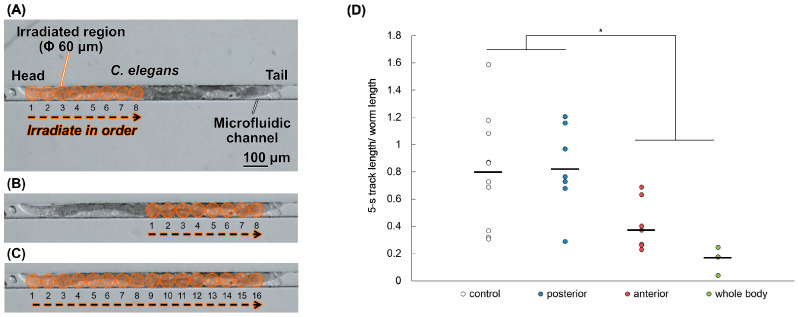
Crawling ability of *C. elegans* immediately after carbon-ion microbeam irradiation. Well-fed adult worms were enclosed in a PDMS microfluidic chip before microbeam irradiation with carbon ions. We used a Φ60-μm micro-aperture (beam exit), and whole- or half-body irradiation was performed by shifting the sample stage of the microbeam irradiation facility at every 60 μm and irradiating each spot in order. Procedures for (**A**) anterior half-body irradiation; for (**B**) posterior half-body irradiation; and for (**C**) whole-body irradiation. After microbeam irradiation, worms were transferred to fresh NGM plates with a bacterial lawn. Crawling of each worm on agar was video-recorded. (**D**) To evaluate motility immediately after carbon-ion microbeam irradiation, the 5-s track length (trajectory) of the center point of the body in crawling on agar was derived from a video based on the procedure shown in Figure 2B and divided by the worm length. Each circle represents the value of 5-s track length per worm length of a single worm and the bar represents mean of each group. The number of the tested worms of each group was as follows: *n* = 11 for the non-irradiated control group (black circle); *n* = 8 for the anterior half-body irradiated group (blue circle); *n* = 9 for the posterior half-body irradiated group (red circle); and *n* = 3 for the whole-body microbeam irradiated group (green circle). The data were analyzed by the Student’s t-test. * indicates *p*-value < 0.05, significantly different from non-irradiated control group (0 Gy) and/or posterior half-body irradiated group. Posterior half-body irradiated group was not significantly different from control group. Anterior half-body irradiated group and whole-body irradiated group were significantly different from not only control group but also posterior half-body irradiated group.

In order to assess tissues that exhibit a radiation response related to locomotion, such as crawling and swimming, regions that affect motor function were investigated using region-specific microbeam irradiation. Even when region-specific irradiation was performed on one point of the head, middle, or tail, there was no effect on locomotion, as noted with whole-body irradiation [28]. If there are tissues that are important for the radiation response, irradiation of that region may cause a reduction in motility similar to that of whole-body irradiation. In order to find such a region, the irradiation region was expanded to the anterior or posterior half of the body. To secure a sufficient irradiation range, we used a micro-aperture (beam exit) of Φ60 μm matched to the adult body width, and irradiation to each spot was performed in order by shifting the sample stage of the microbeam irradiation apparatus with 60 μm step (Figure 8). As shown in Figure 8D, after irradiation with 3 kGy, the 5-s track length/body length for the non-irradiation worms was 0.75, that for worms after whole-body irradiation was 0.15, that for worms after anterior-body irradiation was 0.46, and that for worms after posterior-body irradiation was 0.84. The motility of worms whose anterior half of the body was irradiated was slightly lower than that of worms whose posterior half of the body was irradiated, suggesting that the radiosensitivity of the anterior half of the body is higher than that of the posterior half of the body. Therefore, the anterior half of the body may be more important for locomotion than the posterior half of the body.

## 3. Discussion

Adult *C. elegans* is a model organism resistant to radiation [2], but irradiation with 1 kGy of γ-rays or carbon ion beams reduced crawling (locomotion) [17,19]. In the present study, both crawling and swimming motilities were measured. Immediately after 3-kGy irradiation, both motilities were disturbed. We investigated crawling after irradiation with a single dose of 0.5, 1, 3, or 6 kGy, and both γ-rays and carbon-ion beams reduced motility in a dose-dependent manner (Figure 3). After whole-body irradiation with 6 kGy, the crawling of the worms stopped and was not recoverable (Video S2). Irradiation with 3 kGy significantly reduced the motility of worms (Figure 3). A stronger inhibitory effect was observed for swimming than for crawling (Figure 3 and Figure 6). It has been reported that swimming consumes more energy than crawling [48]. Therefore, the inhibitory effects of radiation were strongly noted on swimming. Consistent with the present study, Krisko et al. [2] reported that 3.5-kGy γ-ray irradiation abolished the stimulus response in approximately 90% of L4 *C. elegans* in liquid media. At 0.5 and 1 kGy, the effects of carbon-ion beam irradiation on the motility of *C. elegans* were higher than those of γ-rays. At sufficiently high doses, such as 3 kGy or more, γ-rays and carbon-ion beams induced similar effects on motility of *C. elegans* (Figure 3).

The biological effects of radiation are affected by the ionization and excitation distribution states of the radiation trajectory. The linear energy transfer (LET) is the amount of energy that an ionizing particle transfers to the material traversed per unit of distance. In general, the higher the LET, the higher the biological effect, and the lower the oxygen effect and modification effect by sensitizers [49]. Typical low-LET radiation includes X-rays and γ-rays. Typical high-LET radiation includes heavy particle radiation and neutron radiation. High-LET radiation causes large, localized DNA damage, which is difficult to repair by cellular repair mechanisms [50,51,52]. In *C. elegans*, high-LET irradiation induces more cell death in the gonads, which are dividing cells, than low-LET irradiation [53]. In contrast, the present study demonstrated similar effects on whole-body locomotion (crawling) of adult *C. elegans* by both high- and low-LET irradiation with doses of over 3 kGy. As the adult worm body consists of the non-dividing somatic cells without nuclear DNA replication, the radiation-induced effects derived from nuclear DNA damage were considered to be small. The radiation effects on motility of *C. elegans* observed may depend on the responses to radiation damage in the cytoplasm such as those of proteins and mitochondria.

We observed swimming after whole-body γ-ray irradiation over time, and found that the motility recovered to approximately 50% of the non-irradiated level 6 h after irradiation with 3 kGy of γ-rays (Figure 6). We previously reported the recovery of motility in *C. elegans* irradiated with 0.5 kGy [19], but the present study revealed that there is a mechanism for recovery of motility even at 3 kGy.

Accumulation of abnormal proteins and organelles, such as mitochondria, may inhibit movement [40,41]. Autophagy is one of the mechanisms by which abnormal proteins and non-functioning mitochondria are degraded [24,39,42]. We previously reported that autophagy is induced by ROS after 7 h in *C. elegans* intestinal cells by the *lgg-1*p::GFP::*lgg-1* reporter method [20]. Using this method, we examined the induction of autophagy by radiation and found that autophagy was also induced after 7 h (Figure 7B). Radiation induced autophagy mainly in the pharynx (head), intestinal tract (anterior body), and motor neurons along the body-wall muscles. A nerve ring with a concentration of neurons wraps around the pharynx; induction of autophagy by 44 mM H_2_O_2_ treatment was previously observed throughout the body [20]. As H_2_O_2_ is produced after irradiation with 3 kGy at room temperature and pH neutrality corresponds to 0.2–0.3 mM [17], the difference between the regions of autophagy induced by irradiation and H_2_O_2_ may be due to the different dosage. In addition, the autophagy observed 7 h after whole-body irradiation disappeared after 28 h (Figure 7C), suggesting that the response was temporary.

The time to recover from motor inhibition and that to induce autophagy were both 6-to-7 h. In addition, autophagy was induced at regions related to motor function. Whether there is a relationship between radiation-induced autophagy and motor recovery is of interest.

In a previous report, spot irradiation of 0.5 kGy to the head, mid-body, or tail had no effect on motor function [28]. Local irradiation of the head including the CNS with 1 kGy reduced motility in crawling temporarily, but a normal pattern of muscle contraction was observed in the non-irradiated body-wall muscle cells from head to tail, even immediately after irradiation [18]. If the inhibition of motor function by radiation reflects a specific region response, similar inhibition should occur after region-specific irradiation. As shown in Figure 8D, stronger effects on crawling were observed in the worms whose anterior half of the body was irradiated than in those whose posterior half of the body was irradiated by 3 kGy. This suggests that the anterior half of the body is an important region for locomotion-related radiation responses. As the CNS is around the pharynx, the restoration of cellular function may lead to the recovery of motility. In the intestine, autophagy is thought to restore the energy-producing function, which may lead the recovery of the motility.

**Figure 9:**
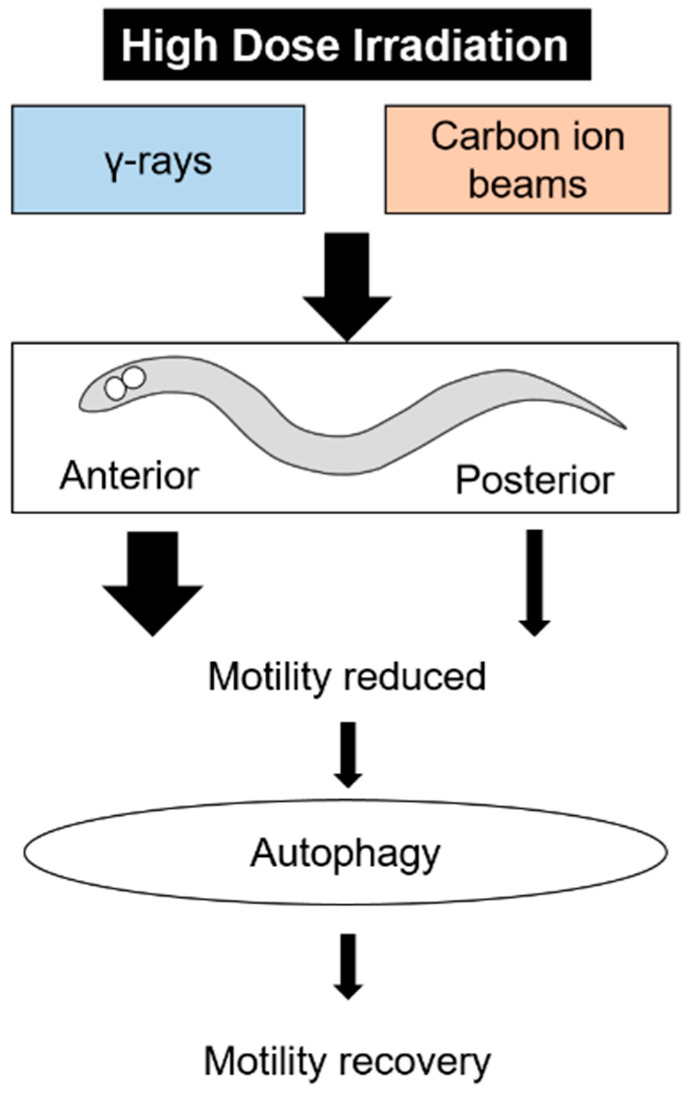
The summary of this study on effects of high-dose irradiation.

This study provided good materials for elucidating the mechanisms of radiation response in locomotion (Figure 9). In space radiation protection, it is important to prevent the effects of radiation on motor functions. This study suggests that there is a recovery mechanism for impaired motor function by radiation. By screening using this experimental system, compounds that promote recovery function can be explored. In addition, it will be useful to focus on genes expressed in the anterior half of the body to search for the response genes. These studies may lead to the development of acquired resistance to radiation. In the future, we would like to elucidate the mechanism of radiation induced autophagy, and its relationship with exercise combining inhibitors and mutants.

## 4. Materials and Methods

### 4.1. Strains and Culture

Wild-type (Bristol N2) and DA2123 [*lgg-1*p::GFP::*lgg-1* + *rol-6*(*su1006*)] *C. elegans* strains [54], and *Escherichia coli* (*E. coli*) strain OP50 were obtained from the Caenorhabditis Genetic Center (The University of Minnesota, Minneapolis, MN, USA). All *C. elegans* hermaphrodites were maintained on 60 mm Petri plates (IWAKI nontreated dish, AGC Techno Glass, Shizuoka, Japan) containing standard NGM spread with *E. coli* OP50 as a food at 20 °C [8]. The NGM consisted of 0.3% (*w*/*v*) NaCl, 0.25% (*w*/*v*) polypeptone, 0.005% (*w*/*v*) cholesterol, 1 mM MgSO_4_, 1 mM CaCl_2_, 25 mM potassium phosphate, pH 6.0, and 0.17% (*w*/*v*) agar. Well-fed adult worms, approximately 3 days after hatching, were used in all experiments.

### 4.2. Whole-Body Irradiation

Whole-body irradiation with γ-rays to *C. elegans* was performed according to a previously described method [17,19]. Briefly, worms on the 60 mm Petri plates containing 10 mL of NGM spread with a bacterial lawn (food) were exposed to ^60^Co γ-rays (32 Gy/min) at 20 °C in the ^60^Co γ-rays irradiation facility at QST-Takasaki. Whole-body broad beam carbon-ion irradiation was performed according to a previously described method [27,29]. Briefly, worms on a 60 mm Petri plate containing 10 mL of NGM spread with a bacterial lawn as food were irradiated by a scan beam of carbon ion (^12^C^5+^,18.3 [MeV/u]) at a dose of 0–6 kGy. Irradiation was carried out at the Takasaki Ion Accelerators for Advanced Radiation Application (TIARA) facility at QST-Takasaki. The LET of the carbon-ion broad beam passing through the worm was approximately 119 keV/μm at the surface of the NGM plate. For all irradiation experiments, non-irradiated control worms were handled in parallel with irradiated worms.

### 4.3. Crawling Analysis

To evaluate the effects of whole-body irradiation with γ-rays or carbon-ion beams on motility, immediately after irradiation, crawling, such as forward motion, backward motion, and turning of the worms on the NGM plates spread with a bacterial lawn was video-recorded (Figure 1 and Figure 2A). A digital camera video-recorder (EX-F1, Casio Computer Co., Ltd., Tokyo, Japan) mounted on a stereomicroscope (SZX7, Olympus Corporation, Tokyo, Japan) was used for video-recording. We first decided the shooting magnification (Figure 2B) to conduct the video-based worm tracking. The crawling was recorded for 10 s. In this condition, the duration available for analysis was confirmed as 5 s, i.e., the non-irradiated control worms moving more rapidly than the irradiated worms, did not frame out for at least 5 s. In the crawling analysis, each worm was needed to be photographed separately. The crawling of each worm was recorded individually in the video. Because it took time to take a movie, the number of worms were limited in order to reduce the error in elapsed time. To obtain accurate results, the number of worms to be measured at one time point was set to 5.

The movies of 5 worms were analyzed using a *C. elegans*-specific image processing software, WormLab^®^4.0 (MBF Bioscience, Williston, VT, USA) [31], and the mean of 5 second track (trajectory) was derived from 5 worms (Figure S1 for 0 h, Figure S2 for 24 h). The main procedure of image processing for worm tracking using WormLab^®^4.0 is as follows: (1) import a movie, (2) set a threshold for binalization of images to extract *C. elegans*, (3) extract the centre line (yellow line in Figure 2B) and centre point (green circle in Figure 2B) of the worm body, (4) repeat procedure (3) for 151 images of a 5 s movie, and finally (5) derive the 5 second track length (green line in Figure 2B) based on data of 151 images, automatically.

In addition, this video-based worm tracking analysis of crawling was applied to evaluate the effects of microbeam irradiation on motility at an individual worm level. The 5 second track length/body length of 5 worms per experiment was calculated and averaged. Then, the normalized value of motility was calculated by dividing the irradiated group by the control group at the same time.

Thirty worms were tested in each condition in 6 trials of γ-rays irradiation (5 worms per condition per trial). Twenty worms were tested in each condition in 4 trials of carbon-ion irradiation (5 worms per condition per trial). Then, statistical analysis was performed.

### 4.4. Swimming Analysis

To further evaluate the effects of whole-body irradiation with γ-rays or carbon-ion beams on motility, swimming of the irradiated worms in S-basal buffer solution (0.59% (*w*/*v*) NaCl, 0.005% (*w*/*v*) cholesterol, and 50 mM potassium phosphate, pH 6.0) was video-recorded. The experimental condition was designed based on a previous study [33,34]. Immediately after irradiation, adult worms were incubated on NGM plates spread with a bacterial lawn at 20 °C for 0, 6, 12, or 24 h. As shown in Figure 5A, after incubation, irradiated worms were washed using S-basal buffer solution twice and transferred to a fresh NGM plate without a bacterial lawn. Immediately after transfer, 3 mL or more of S-basal buffer solution was injected into the plate and swimming worms were video-recorded. The number of head swings of each worm for 20 s was counted based on the movie (Figure 5B) and the values of 10 worms were averaged. Three independent irradiation experiments and swimming assays were conducted.

### 4.5. Fluorescence Microscopy Observation

To detect the induction of autophagy, the DA2123 [*lgg-1*p::GFP::*lgg-1* + *rol-6*(*su1006*)] strain was purchased from the Caenorhabditis Genetic Center. After irradiation, adult DA2123 worms were incubated on an NGM plate spread with a bacterial lawn for 0–28 h at 20 °C. After incubation, worms were paralyzed using 0.5 M sodium azide and mounted on an agar pad. Then, GFP fluorescence from the worms was observed by confocal microscopy (FV1000, Olympus Corporation, Tokyo, Japan) using a 488 nm argon laser. To identify the expression site, we conducted in the least fluorescent setting.

### 4.6. Region-Specific Carbon-Ion Microbeam Irradiation

To investigate the involvement of specific regions of the body, we employed the targeted-microbeam irradiation technique. Carbon-ion (^12^C^5+^) particles were accelerated by an AVF cyclotron at TIARA and with the method previously described [19,30].

*C. elegans* were immobilized for microbeam irradiation according to a previously described method [19]. Briefly, to inhibit the movement of worms during irradiation without the use of anesthesia, washed worms were enclosed in straight channels of a PDMS microfluidic chip (Worm Sheet IR, Biocosm Inc., Hyogo, Japan) before carbon-ion microbeam irradiation.

The LET of the carbon ions at the surface of the microfluidic chip (depth from the surface of a sample = 125 μm) was approximately 106 keV/μm and that at the bottom of the microfluidic channel (depth from the surface of a sample = 195 μm) was approximately 110 keV/μm. Therefore, the track-averaged LET of carbon ions passing through a worm enclosed in a microchannel was approximately 108 keV/μm. From this value we calculated the number of ions that would give the desired absorbed dose to the targeted region. As a practical case, 600,000 particles correspond to a dose of 3 kGy at a micro-aperture area of Φ60 μm.

In order to target an area the same size as the body width of an adult worm, we used a Φ60-μm micro-aperture (beam exit) [30] for anterior half-body irradiation (Figure 8A), posterior half-body irradiation (Figure 8B), and whole-body irradiation (Figure 8C). We newly established a half-body irradiation method, in which we irradiated each spot in order by shifting the sample stage of the microbeam irradiation apparatus at every 60 μm (irradiation range), as shown in Figure 8A–C. Immediately after irradiation, the worms were washed in a drop of wash buffer solution (0.05% (*w*/*v*) gelatine, 1 mM MgSO_4_, 1 mM CaCl_2_, and 5 mM potassium phosphate, pH 6.0), and transferred one by one to fresh NGM plates with OP50 using a platina picker (Worm Stuff Worm Pick, Genesee Scientific Corporation, San Diego, CA, USA). Thereafter, we conducted the same crawling assay described above in the case of whole-body irradiation. For all irradiation experiments, non-irradiated control worms were handled in parallel with irradiated worms.

### 4.7. Statistical Analysis

Statistical analysis was performed on more than 3 independent experiments of whole-body irradiation. The significance of differences was examined using the Student’s *t* test. *p* < 0.05 was considered significant. All statistical analyses were performed using Microsoft Excel software (Microsoft, Redmond, WA, USA).

## 5. Conclusions

This study showed that motility in adult *C. elegans* was reduced by whole-body irradiation with high-dose γ-rays and carbon-ion beams. The decreased motility after whole-body irradiation partially recovered. Anterior half-body irradiation by carbon-ion microbeams decreased motility. Autophagy was induced during the recovery of decreased motility after whole-body irradiation (Figure 9). This study provided good materials for elucidating the mechanisms of radiation response in motility. Autophagy may be involved in the restoration of motility through the elimination of damaged components. We will investigate the detailed mechanisms of autophagy and motility recovery in future studies.

## Data Availability

Not applicable.

## Supplementary Materials

The following are available online at https://www.mdpi.com/article/10.3390/ijms22189810/s1, Figure S1: Crawling ability of worms immediately after whole-body irradiation with different doses of γ-rays or carbon-ion beams, Figure S2: Crawling ability of worms 24 h after whole-body irradiation with different doses of γ-rays or carbon-ion beams, Video S1: Typical crawling of non-irradiated control worm on agar for 5-s duration, and Video S2: Typical crawling of worms on agar for 5-s duration after whole-body irradiation with 6 kGy of γ-rays.

## References

[1] Fry R.J.M., Hall E.J. (1995). Radiobiology for the radiologist. Radiat. Res..

[2] Krisko A., Leroy M., Radman M., Meselson M. (2012). Extreme anti-oxidant protection against ionizing radiation in bdelloid rotifers. Proc. Natl. Acad. Sci. USA.

[3] Daly M.J. (2012). Death by protein damage in irradiated cells. DNA Repair.

[4] Filomeni G., de Zio D., Cecconi F. (2015). Oxidative stress and autophagy: The clash between damage and metabolic needs. Cell Death Differ..

[5] Morinaga H., Yonekura S.I., Nakamura N., Sugiyama H., Yonei S., Zhang-Akiyama Q.M. (2009). Purification and characterization of *Caenorhabditis elegans* NTH, a homolog of human endonuclease III: Essential role of N-terminal region. DNA Repair.

[6] Oh S.-I., Park J.-K., Park S.-K. (2015). Lifespan extension and increased resistance to environmental stressors by N-Acetyl-L-Cysteine in *Caenorhabditis elegans*. Clinics.

[7] Michaelidesová A., Konířová J., Bartůněk P., Zíková M. (2019). Effects of radiation therapy on neural stem cells. Genes.

[8] Kuzmic M., Galas S., Lecomte-Pradines C., Dubois C., Dubourg N., Frelon S. (2019). Interplay between ionizing radiation effects and aging in *C. elegans*. Free Radic. Biol. Med..

[9] Heselich A., Frieß J.L., Ritter S., Benz N.P., Layer P.G., Thielemann C. (2018). High LET radiation shows no major cellular and functional effects on primary cardiomyocytes in vitro. Life Sci. Space Res..

[10] Brenner S. (1974). The genetics of *Caenorhabditis elegans*. Genetics.

[11] Kimble J., Hirsh D. (1979). The postembryonic cell lineages of the hermaphrodite and male gonads in *Caenorhabditis elegans*. Dev. Biol..

[12] Sulston J.E., Horvitz H.R. (1977). Post-embryonic cell lineages of the nematode, *Caenorhabditis elegans*. Dev. Biol..

[13] Dhakal R., Yosofvand M., Yavari M., Abdulrahman R., Schurr R., Moustaid-Moussa N., Moussa H. (2021). Review of biological effects of acute and chronic radiation exposure on *Caenorhabditis elegans*. Cells.

[14] Sugimoto T., Dazai K., Sakashita T., Funayama T., Wada S., Hamada N., Kakizaki T., Kobayashi Y., Higashitani A. (2006). Cell cycle arrest and apoptosis in *Caenorhabditis elegans* germline cells following heavy-ion microbeam irradiation. Int. J. Radiat. Biol..

[15] Sakashita T., Suzuki M., Hamada N., Shimozawa Y., Shirai-Fukamoto K., Yokota Y., Hamada-Sora S., Kakizaki T., Wada S., Funayama T. (2012). Behavioral resistance of *Caenorhabditis elegans* against high-LET radiation exposure. Biol. Sci. Space.

[16] Ishii N., Suzuki K. (1990). X-ray inactivation of *Caenorhabditis elegans* embryos or larvae. Int. J. Radiat. Biol..

[17] Johnson T.E., Hartman P.S. (1988). Radiation effects on life span in *Caenorhabditis elegans*. J. Gerontol. Biol. Sci..

[18] Sakashita T., Hamada N., Ikeda D.D., Suzuki M., Yanase S., Ishii N., Kobayashi Y. (2008). Locomotion—Learning behavior relationship in *Caenorhabditis elegans* following γ-ray irradiation. J. Radiat. Res..

[19] Suzuki M., Soh Z., Yamashita H., Tsuji T., Funayama T. (2020). Targeted central nervous system irradiation of *Caenorhabditis elegans* induces a limited effect on motility. Biology.

[20] Suzuki M., Sakashita T., Yanase S., Kikuchi M., Ohba H., Higashitani A., Hamada N., Funayama T., Fukamoto K., Tsuji T. (2009). Effects of ionizing radiation on locomotory behavior and mechanosensation in *Caenorhabditis elegans*. J. Radiat. Res..

[21] Moriwaki T., Yamasaki A., Zhang-Akiyama Q.M. (2018). ATM induces cell death with autophagy in response to H_2_O_2_ specifically in *Caenorhabditis elegans* nondividing cells. Oxid. Med. Cell. Longev..

[22] Mizushima N. (2007). Autophagy: Process and function. Genes Dev..

[23] Orrenius S., Kaminskyy V.O., Zhivotovsky B. (2013). Autophagy in toxicology: Cause or consequence?. Annu. Rev. Pharmacol. Toxicol..

[24] Morishita H., Mizushima N. (2019). Diverse cellular roles of autophagy. Annu. Rev. Pharmacol. Toxicol..

[25] Galluzzi L., Pietrocola F., Levine B., Kroemer G. (2014). Metabolic control of autophagy. Cell.

[26] Vermezovic J., Stergiou L., Hengartner M.O., d’Adda di Fagagna F. (2012). Differential regulation of DNA damage response activation between somatic and germline cells in *Caenorhabditis elegans*. Cell Death Differ..

[27] Hou J., Han Z.P., Jing Y.Y., Yang X., Zhang S.S., Sun K., Hao C., Meng Y., Yu F.H., Liu X.Q. (2013). Autophagy prevents irradiation injury and maintains stemness through decreasing ROS generation in mesenchymal stem cells. Cell Death Dis..

[28] Funayama T. (2019). Heavy-ion microbeams for biological science: Development of system and utilization for biological experiments in QST-takasaki. Quantum Beam Sci..

[29] Suzuki M., Hattori Y., Sakashita T., Yokota Y., Kobayashi Y., Funayama T. (2017). Region-specific irradiation system with heavy-ion microbeam for active individuals of *Caenorhabditis elegans*. J. Radiat. Res..

[30] Suzuki M., Sakashita T., Hattori Y., Yokota Y., Kobayashi Y., Funayama T. (2018). Development of ultra-thin chips for immobilization of *Caenorhabditis elegans* in microfluidic channels during irradiation and selection of buffer solution to prevent dehydration. J. Neurosci. Methods.

[31] Suzuki M., Sakashita T., Funayama T. (2019). Immobilization of live *Caenorhabditis elegans* individuals using an ultra-thin polydimethylsiloxane microfluidic chip with water retention. J. Vis. Exp..

[32] Roussel N., Sprenger J., Tappan S.J., Glaser J.R. (2014). Robust tracking and quantification of *C. elegans* body shape and locomotion through coiling, entanglement, and omega bends. Worm.

[33] Moriwaki T., Kato S., Kato Y., Hosoki A., Zhang-Akiyama Q.-M. (2013). Extension of lifespan and protection against oxidative stress by an antioxidant herb mixture complex (KPG-7) in *Caenorhabditis elegans*. J. Clin. Biochem. Nutr..

[34] Gaffney C.J., Bass J.J., Barratt T.F., Szewczyk N.J. (2014). Methods to assess subcellular compartments of muscle in *C. elegans*. J. Vis. Exp..

[35] Hosoki A., Yonekura S.-I., Zhao Q.-L., Wei Z.-L., Takasaki I., Tabuchi Y., Wang L.-L., Hasuike S., Nomura T., Tachibana A. (2012). Mitochondria-targeted superoxide dismutase (SOD2) regulates radiation resistance and radiation stress response in HeLa Cells. J. Radiat. Res..

[36] Yamamori T., Ike S., Bo T., Sasagawa T., Sakai Y., Suzuki M., Yamamoto K., Nagane M., Yasui H., Inanami O. (2015). Inhibition of the mitochondrial fission protein dynamin-related protein 1 (Drp1) impairs mitochondrial fission and mitotic catastrophe after x-irradiation. Mol. Biol. Cell.

[37] Kam W.W., Banati R.B. (2013). Effects of ionizing radiation on mitochondria. Free Radic. Biol. Med..

[38] Rao R.V., Bredesen D.E. (2004). Misfolded proteins, endoplasmic reticulum stress and neurodegeneration. Curr. Opin. Cell Biol..

[39] Palikaras K., Lionaki E., Tavernarakis N. (2018). Mechanisms of mitophagy in cellular homeostasis, physiology and pathology. Nat. Cell Biol..

[40] Pickles S., Vigié P., Youle R.J. (2018). Mitophagy and quality control mechanisms in mitochondrial maintenance. Curr. Biol..

[41] Kirstein-Miles J., Morimoto R.I. (2010). *Caenorhabditis elegans* as a model system to study intercompartmental proteostasis: Interrelation of mitochondrial function, longevity, and neurodegenerative diseases. Dev. Dyn..

[42] Taferner A., Pircher H., Koziel R., von Grafenstein S., Baraldo G., Palikaras K., Liedl K.R., Tavernarakis N., Jansen-Dürr P. (2015). FAH domain containing protein 1 (FAHD-1) is required for mitochondrial function and locomotion activity in *C. elegans*. PLoS ONE.

[43] Qi Y., Qiu Q., Gu X., Tian Y., Zhang Y. (2016). ATM mediates spermidine-induced mitophagy via PINK1 and Parkin regulation in human fibroblasts. Sci. Rep..

[44] Meesungnoen J., Benrahmoune M., Filali-Mouhim A., Mankhetkorn S., Jay-Gerin J.P. (2001). Monte Carlo calculation of the primary radical and molecular yields of liquid water radiolysis in the linear energy transfer range 0.3–6.5 keV/μm: Application to 137Cs Gamma rays. Radiat. Res..

[45] Meesungnoen J., Jay-Gerin J.P. (2005). High-LET radiolysis of liquid water with ^1^H^+^, ^4^He^2+^, ^12^C^6+^, and ^20^Ne^9+^ ions: Effects of multiple ionization. J Phys. Chem. A.

[46] Moriwaki T., Kato Y., Nakamura C., Ishikawa S., Zhang-Akiyama Q.M. (2015). A novel DNA damage response mediated by DNA mismatch repair in *Caenorhabditis elegans*: Induction of programmed autophagic cell death in non-dividing cells. Genes Cancer.

[47] Zhang H., Chang J.T., Guo B., Hansen M., Jia K., Ková A.L., Kumsta C., Lapierre L.R., Legouis R., Lin L. (2015). Guidelines for monitoring autophagy in *Caenorhabditis elegans*. Autophagy.

[48] Palmisano N.J., Meléndez A. (2016). Detection of autophagy in *Caenorhabditis elegans* using GFP::LGG-1 as an autophagy marker. Cold Spring Harb. Protoc..

[49] Laranjeiro R., Harinath G., Burke D., Braeckman B.P., Driscoll M. (2017). Single swim sessions in *C. elegans* induce key features of mammalian exercise. BMC Biol..

[50] Hirayama R. (2014). Mechanism of oxygen effect for photon and heavy-ion beams. Jpn. J. Med Phys..

[51] Hada M., Alexandros G.G. (2008). Formation of clustered DNA damage after high-LET irradiation: A review. J. Radiat. Res..

[52] Terato H., Ide H. (2004). Clustered DNA damage induced by heavy ion particles. Biol. Sci. Space.

[53] Takanami T., Zhang Y., Aoki H., Abe T., Yoshida S., Takahashi H., Horiuchi S., Higashitani A. (2003). Efficient repair of DNA damage induced by heavy ion particles in meiotic prophase I nuclei of *Caenorhabditis elegans*. J. Radiat. Res..

[54] Kang C., You N.J., Avery L. (2007). Dual roles of autophagy in the survival of *Caenorhabditis elegans* during starvation. Genes Dev..

