# Supplementary material for: High-Dose Irradiation Inhibits Motility and Induces Autophagy in Caenorhabditis elegans
Source: Int J Mol Sci. 2021 Sep 10;22(18):9810. doi: 10.3390/ijms22189810 (PMC8467272; doi:10.3390/ijms22189810)
Supplement: Supplementary file 1 [file ijms-22-09810-s001.zip › Supplementary Materials.pdf]

Supplementary Materials

High-Dose Irradiation Inhibits Motility and Induces Autophagy in *Caenorhabditis elegans*

Akira Yamasaki [1,2], Michiyo Suzuki [2,*], Tomoo Funayama [2], Takahito Moriwaki [1,3], Tetsuya Sakashita [2], Yasuhiko Kobayashi [2], and Qui-Mei Zhang-Akiyama [1,*]

[1] Laboratory of Stress Response Biology, Graduate School of Science, Kyoto University, Kitashirakawa-Oiwakecho, Sakyo-ku, Kyoto 606-8502, Japan

[2] Department of Radiation-Applied Biology Research, Takasaki Advanced Radiation Research Institute, National Institutes for Quantum and Radiological Science and Technology (QST-Takasaki), 1233 Watanuki, Takasaki 370-1292, Japan

[3] Department of Pathophysiology and Metabolism, Kawasaki Medical School, Okayama, 701-0192, Japan

Correspondence:

* Qiu-Mei Zhang-Akiyama, Ph.D.

Laboratory of Stress Response Biology, Graduate School of Science, Kyoto University,

Kitashirakawa-Oiwakecho, Sakyo-ku, Kyoto 606-8502, Japan

Email: qmzhang@kingyo.zool.kyoto-u.ac.jp; Tel.: +81-(0)75-753-4097; Fax: +81-(0)75-753-4087

* Michiyo Suzuki, Ph.D.

Department of Radiation-Applied Biology Research, Takasaki Advanced Radiation Research Institute, National Institutes for Quantum and Radiological Science and Technology (QST-Takasaki),

1233 Watanuki, Takasaki 370-1292, Japan

Email: suzuki.michiyo.@qst.go.jp; Tel.: +81-(0)27-346-9114; Fax: +81-(0)27-346-9353

Supplementary Figures

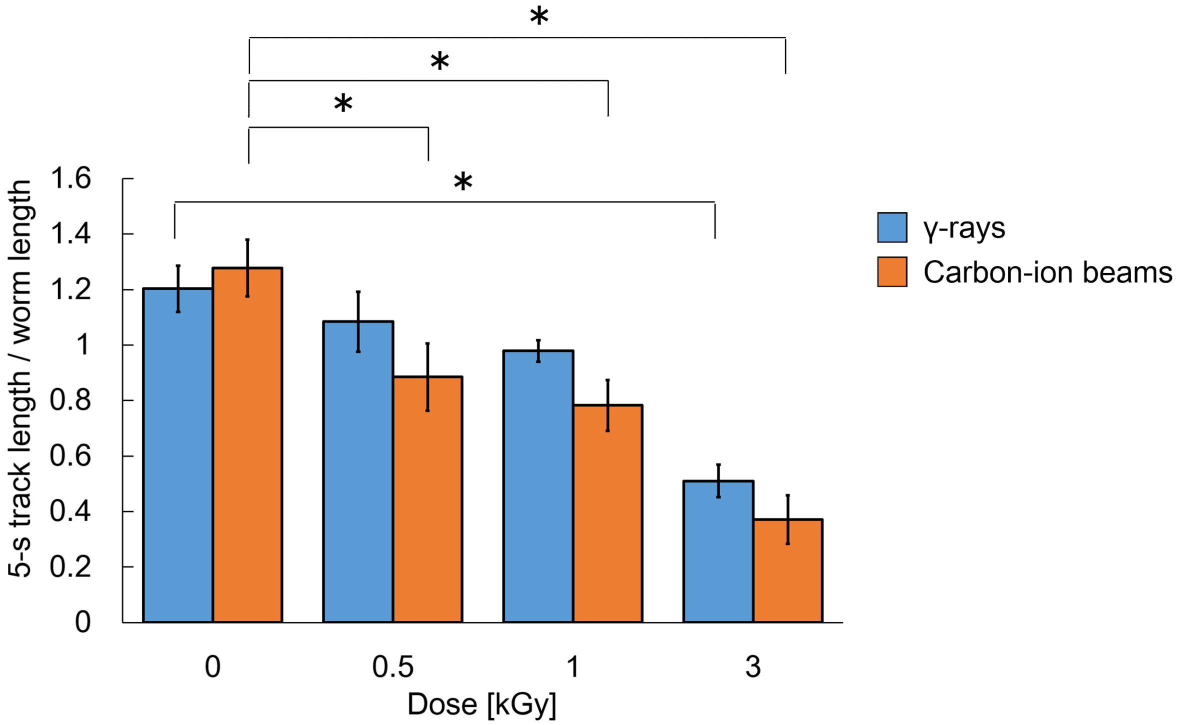

Figure S1. Crawling ability of worms immediately after whole-body irradiation with different doses of γ-rays or carbon-ion beams. To evaluate motility of the irradiated worms, trajectories in crawling for 5-s duration were extracted and divided by the worm length, and the values of 5 worms were averaged at each condition every experiment. For each dose of radiation, 6 trials for γ-rays and 4 trials for carbon-ion broad beams were conducted. Error bar indicates SEM. The data was analyzed by the Student's *t*-test. * indicates p-value < 0.05, significantly different one compared with the 0 kGy.

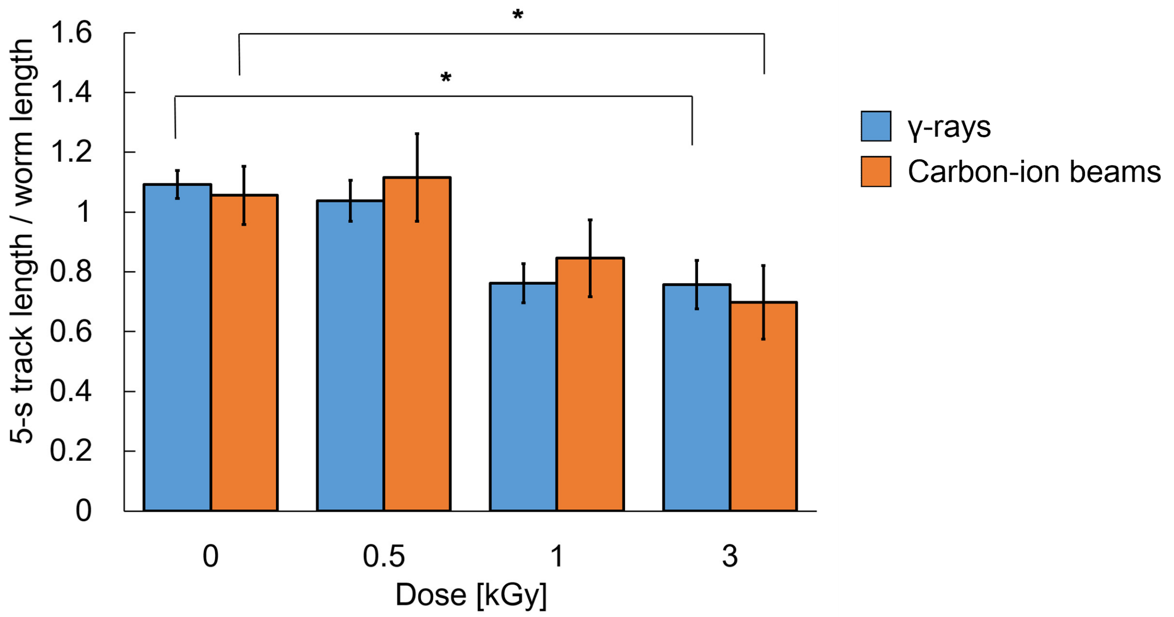

Figure S2. Crawling ability of worms 24 hours after whole-body irradiation with different doses of γ-rays or carbon-ion beams. To evaluate motility of the irradiated worms, trajectories in crawling for 5-s duration were extracted and divided by the worm length, and the values of 5 worms were averaged at each condition every experiment. For each dose of radiation, 6 trials for γ-rays and 4 trials for carbon-ion broad beams were conducted. Error bar indicates SEM. The data was analyzed by the Student's *t*-test. * indicates p-value < 0.05, significantly different one compared with the 0 kGy.

Video S1. Typical crawling of non-irradiated control worm on agar for 5-s duration.

Original video of non-irradiated worm analyzed in Figure. 2B.

Video S2. Typical crawling of worms on agar for 5-s duration after whole-body irradiation with 6 kGy of γ-rays.

Each movie represents same three worms irradiated with 6 kGy (A) immediately after irradiation (0 hour), or (B) 24 hours after irradiation.
